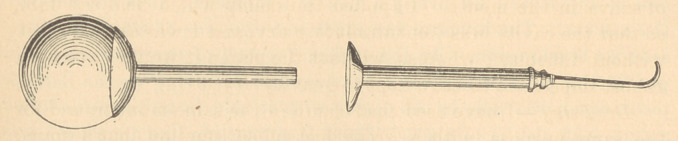# New York Odontological Society

**Published:** 1890-10

**Authors:** 

**Affiliations:** New York Odontological Society


### NEW YORK ODONTOLOGICAL SOCIETY.

The New York Odontological Society held its regular monthly meeting Tuesday evening, May 20, 1890, in the New York Academy of Medicine, No. 12 West Thirty-first Street. The President, Dr. J. Morgan Howe, in the chair.

### INCIDENTS OF OFFICE PRACTICE AND CASUAL COMMUNICATIONS.

*Dr. S. G. Perry.*—There is no workman in the world who should have such delicacy of touch as the dentist, and in order to have that he must have soft fingers and not be in the habit of handling harsh instruments. I have had great pleasure in the use of wooden handles of different forms. I have had them made of various sizes and styles. I have some here made of Stubb's wire and put into the wood nearly the whole length of the handle. They have no ferrules. The wire inside the handle gives great firmness and stiffness to the instrument. These handles are very small, for the reason that the points are very delicate. The size of the handle should always be proportionate to the size of the instrument itself. They have a feeling of softness and delicacy in the hand that cannot be had in the steel instrument. In fact they are simply steel instruments covered with wood. I am having some made that will be covered with vulcanized black rubber. Instrument-makers have a great fancy for making their instruments "pretty," and just in the proportion that they make them pretty by knurling and milling they make them unfit for use. I think the men who use tools should have them as smooth and soft in feeling as they can be made, and if the instrument can be partly filled out with wood it will give delicacy of touch and lightness also.

I wish to show here a very delicate mandrel for holding extremely small sand-paper disks. In order to change the disk quickly one must have some means of holding either the nut or the screw. Many years ago the S. S. White Company made for me a small mandrel with a screw-driver that engaged and held the screw. These could be quickly changed, but in time the square holes in the ends of the screws became worn, and as the disks were put on the screw instead of on the mandrel they would often drop off the end of the screw-driver.

In these I put the disks on the mandrels, and I have here a screw-driver that slips over and holds the nut with unerring certainty. The screw-driver being magnetized, the nut is held firmly in place. The mandrel is made complete from a piece of No. 42 gauge wire. It can be cut by the bur-cutting machinery to be as delicate as the burs of the smallest size. Disks about a quarter of an inch in diameter, mounted on these slender mandrels, are very useful in finishing proximate fillings, as well as all of those near the gum on the labial and buccal surfaces.

*Dr. Jarvie.*—I wish every gentleman here could see the device I saw in Albany, last week, for holding sand-paper disks. It enables one to adjust and fix firmly a disk in a moment. It is not yet on the market. The inventor has made but two, one of which he had sent to Washington. By the device I speak of the disk may be put in the palm of the hand, the mandrel revolving in the hand-piece, touch the hole in the centre with it, and in an instant—like magic—the disk is in place. There is something like a cork-screw on the end of the mandrel; not a wire cork-screw, but one cut out of a piece of steel, having perhaps two turns, ending at a shoulder; there is a little lug that the disk slips over and which prevents it from returning if the mandrel is reversed. It is the simplest thing in the world.

*Dr. Meriam.*—I do not know how many of the gentlemen present remember the exhibit at the International Medical Congress. I studied it pretty well, and thought we would some time have an exhibit in Massachusetts, made up on that model. Two years ago I was able to aid in arranging one. We tried to make the exhibit not only one of appliances, but of appliances exhibited by actual producers, and materials that could be traced to a source; among the exhibitors was the Davidson Rubber Company, of Boston, —the first rubber company that ever exhibited at a dental meeting, —exhibiting its rubber dam; and since then we have arranged with a maker of surgical instruments, Goldthwaite & Company, Washington Street, Boston, to keep this rubber for us. I believe this to be almost the only rubber sold in the name of the manufacturer, and by the pound. I have brought some samples with me. The wholesale cost is, I am informed, the highest of any rubber dam made.

*Dr. Kingsley.*—I wonder if I am the only one in the society who has ever been bothered with syringes. I think that the best syringe for washing out a cavity which is being prepared, is a syringe with a rubber bulb,—and I have found that if the outlet is as small as I would like to have it, so as not to deluge the mouth with a sudden flow, too much time is consumed in filling the bulb.

I am not a very patient man while waiting for a bulb to fill, and seconds seem minutes sometimes. What I would really like would be a syringe with a very small outlet, which would give force without discharging such a quantity of water, particularly when I have the rubber dam on a tooth. I am not satisfied with blowing the chips out, I want to wash out the cavity, but I don't want to deluge the cavity and the rubber dam. It takes but a very little water, if there be force enough. To overcome the difficulty I conceived a double-tube syringe. The tube, which is attached to the bulb, slides into the tube which carries the point, and fits so accurately that there is no leakage, or if there should prove to be any, a small washer of sheet rubber corrects it. The point of this is very fine and will give a jet of great force. I found, by experiment, that to fill the bulb by sucking it through the point required over one minute, while detached it would fill quick as a flash.

I have looked around and have not found anything like it in the market. The nearest thing to it that I saw was one which would unscrew. The bulb is filled and screwed up again, and in the mean time more time has been lost than by the old-fashioned way. If this of mine is found to be as convenient to others as it is to me it will be very valuable.

*Dr. C. A. Woodward.*—It will be remembered that two or three months ago, in one of our meetings, Dr. Jarvie presented a model of some superior teeth, and stated that he believed that one of the central incisors had been considerably shortened. Some who were present were of the opinion that, instead of that one shortening, the others had elongated. I have here a model of a case in which I believe the tooth has shortened nearly the sixteenth of an inch, and the others have not elongated. The teeth are in a normal, healthy condition, and the shortening has been going on for some four or five years.

One thing more. Some two years ago I had made a set of burs for trimming the roots of teeth. I have had a great deal of trouble in trimming teeth without lacerating the gums. Most of the burs in the market are course and cut too rapidly, and the shapes did not suit me. I have a set of burs here of an oval shape, or rather more like a saucer. They are not course, neither are they very fine. I find that I can trim roots with them more rapidly than with any other burs that I have used, and without injuring the gums.

*Dr. S. F. Howland.*—At a recent meeting Dr. Howe exhibited a clamp for retaining the rubber dam while filling labial cervical cavities. Dr. Howe kindly loaned me one of them, and I have used it with a great deal of satisfaction. My first trial was on a lower cuspid having a very large cavity, and there was an abundant flow of saliva in the mouth. I applied the clamp which held the dam, so that the cavity was kept absolutely dry, and I was able to fill it without difficulty; whereas, without the clamp it would have been almost impossible to have kept the cavity dry.

*Dr. Perry.*—I have used that clamp in the same manner, and for the same purpose, with a great deal of satisfaction, but I found there was difficulty sometimes in adjusting the holder, and I have a number of times tied that little outer disk to the tooth itself, instead of using the clamp, and it worked admirably. I would like to ask the president if he has used it in that manner.

*President Howe.*—Yes, tying with thread or silk is a very good way of holding the dam-retainer in place on a tooth. The clamp is not an essential part of the device, it is only one means of holding the retainer in place.

*Dr. Perry.*—I have had such good results in tying that it seemed to me hardly possible to improve upon that method. I can hardly think of a case that I could not control completely by tying that little device to the tooth. I think it is a most valuable acquisition.

*Dr. Meriam.*—I wish to say a word about that clamp. I filled the other day a distal cavity, extending above the gum, in a cuspid tooth, the first bicuspid having been lost; always a very annoying cavity. I had a chance to use the plate, and tied it on, doing away with the holder, and had very excellent results indeed. One especial advantage is, that the face-plate lies so close down to the tooth that the cavity can be filled without the necessity of reaching through any great distance.

*Dr. S. H. McNaughton.*—A few days ago I received a note from your president asking about a preparation I use for devitalizing pulps, and adding that he should like to have me present the formula of it to this society. The formula is—

Thymol, 24 grains;<br>

Camphor, 12 grains;<br>

Arsenious acid, 3 grains.

It acts much as other "nerve pastes" do, except that it hardens and toughens the pulps and makes its removal easier. And there is no decomposition or disintegration of the pulp, even if it should remain in the tooth for three or four weeks.

The devitalizing of the pulp is usually accomplished in from two to four days. If one or two applications do not destroy the pulp, then I add a little salicylic acid, which tends to soften and dissolve it; but, by using the salicylic acid, one of the very best qualities of this preparation—that of preserving the pulp from disintegration—is lost. If, after the application of the arsenic, there is severe toothache, I apply to the pulp (without washing or syringing out, and being careful to avoid pressure) another preparation, the formula of which is—

Camphor, 60 grains;
Tannic acid, 30 grains;
Alcohol, 1 ounce.

This will nearly always prove effectual, but may require a half-hour, and the action of the arsenious acid appears to be continued under it. The camphor coagulates and makes a firm plug.

*Dr. Bogue.*—I have been transplanting three or four teeth lately, and one case was to me something unusual. One of my professional friends had sent a patient to Dr. Hasbrouck to have the roots of a bicuspid taken out; and the successful extraction of those roots necessitated the removal of quite a depth of alveolus. Four days later this gentleman was at my office with his sister, and asked if anything could be put in there. I said yes, I can put a tooth in, but it will fall out again, probably; but said he, "Suppose it does; let us try." There was a tooth which had been lying in my drawer nearly six years, and it was put in and tied with silk, and it has never given a moment's trouble. That was done three weeks ago. The absence of periosteum and the absence of alveolus was to me something unusual; altogether so. The use of a tooth that had been lying in my drawer that length of time, and supported only by soft tissue, was purely experimental. We could hardly hope for good results.

*Dr. Atkinson.*—Is the color of the tooth changed to the natural color of the other teeth?

*Dr. Bogue.*—It is still lighter than his own.

*Dr. Atkinson.*—Is the lightness due to drying?

*Dr. Bogue.*—No; I think it is lighter naturally. Of course the root is filled with oxychloride, and a cavity with gold.

*Dr. Kingsley.*—Is it still tied in?

*Dr. Bogue.*—No; the string was cut a week ago.

*Dr. Kingsley.*—That beats my case. I have one that has been in for five years, but it is still tied.

*Dr. Jarvie.*—It may be in because it is tied.

*Dr. Kingsley.*—That is it exactly.

*Dr. Bogue.*—Mr. President, the small boy who was operated upon here by Dr. Curtis, a month or two ago, nitrous oxide being applied to obtund the sensibility of the tooth, complained of a great deal of pain later on, and I corresponded a little with Dr. Curtis in regard to it. He thinks the pain arose from pressure upon the pulp. The boy thinks not, however. He suffered all night, and the next day the gutta-percha was taken out and veratrine put in, and the excavation completed. Whatever the theories may be as to the effects of veratrine, hot air, or nitrous oxide, the boy said that under the veratrine he stood the excavating with perfect ease. Since then there has been no pain whatever; but up to Wednesday afternoon following his treatment there was constant pain from the time he left this room, not quite twenty-four hours. Dr. Curtis's idea is that the pain resulted from pressure. I mention it because we want to know both sides of the question as far as we can.

*The President.*—If there is nothing further to be presented under this head, we will listen to the paper of the evening, by Dr. Charles B. Atkinson.

Dr. Charles B. Atkinson here read a paper entitled, "Observations on Preparation, Discussion, Prevention, Reparation, Association, and Publication as applied to Dentistry."

(For Dr. Atkinson's paper, see page 591).

*The President.*—Gentlemen, you have a wide field open for discussion in the subject that Dr. Atkinson has presented to you in his very suggestive paper. Since the beginning of the meeting I have received a letter from Dr. Bödecker which bears upon one point of the paper. The secretary will kindly read that now.

The secretary read the following communication from Dr. C. F. W. Bödecker:

"60 East Fifty-eighth Street, May 20, 1890.

"Dr. J. M. Howe:

"My dear Sir,—I have read the abstract which you gave me of the paper Dr. C. B. Atkinson is announced to read before your society this evening.

"I am very sorry that I am unable to be with you this evening, as the paper contains many points of great interest. I have frequently considered the feasibility of paragraph No. 21, and am of the opinion that if the dental profession possessed a home in which all dental meetings and clinics could be held, and in which home there was a library, a chemical, histological, and mechanical laboratory, where all dentists who are willing to do something for the advancement of the profession could assemble and receive information or instruction, that such a house would add more towards the advancement of our specialty than anything else. Our profession ought to have a home, which must be independent of manufacturing houses, as well as individual dental societies. I would therefore kindly ask you to make the following proposition to the Odontological Society for me this evening.

"Although I am not rich and have to work for the money as all of us do, yet, for the love of my profession, I am willing to give five thousand dollars ($5000) towards a fund to build a home for the dental profession. I will, however, make this donation under the condition that I, or my wife Wilhelmina, as long as either of us are alive, shall receive an income from the above five thousand dollars amounting to five per cent. per annum. After the death of myself and wife, this money shall become the property of the dental profession. I am willing to deliver this money as soon as twenty thousand dollars have been subscribed for the above-named purpose, after sixty days' previous notice.

"Perhaps the Odontological Society and the First District Dental Society may each nominate some member to form a committee into whose hands this matter might be placed for further consideration.

"Very truly yours,

"(Signed.) C. F. W. Bödecker."

*The President.*—Gentlemen, we will proceed with the discussion of Dr. Atkinson's paper; and at the close of the discussion any motion which any one present wishes to make in relation to Dr. Bödecker's very liberal proposition will be in order.

*Dr. J. O. Flower.*—Mr. President, if I may take the liberty of speaking, the article that Dr. Atkinson has read, supported by Dr. Bödecker's communication, I think is one of the best things that has ever been brought forward. I came here this evening on invitation of Dr. Atkinson, and I am so well pleased that I will state that if this plan is adopted I will make a liberal donation towards the proposed dental home. Although I am not in this society, I have always been received very well at the college here and by the different members of the profession in this city, and if they conclude to make a home for the improvement of dentistry, I will donate a liberal amount of money for the purpose.

*Dr. Jarvie.*—There are so many points covered by this paper that it is really difficult to select any of them to speak upon without a feeling that others are being neglected. There are three points that especially commend themselves to me. In the first place, I want to emphasize especially my approval of the plan of sending copies or synopses of papers to a number of members of the society before the meeting at which they are to be read is held. Many times a paper of great value is read at our meetings, but of such a character that no one feels like discussing or having much to say upon it, consequently the value of the paper for that evening at least is lost; whereas, if a synopsis had been sent to members, they would have had time to study it and prepare their thoughts, and be able to give expression to them at the meeting. I think it is a subject that is worthy of being taken into consideration at this time. How shall we enhance the value of our dental meetings? We have adopted, apparently, a fixed plan at our meetings; and I think if we can get out of it we ought to do so. It is almost impossible to be one of a gathering of even half a dozen dentists, and exchange views, without each one carrying away something of benefit; but, nevertheless, I think we hold a great many meetings where our time is largely lost; and perhaps if a different system had been adopted we might have considered the time very well spent. We often have papers read and subjects brought up for discussion, and we talk a long time upon them without arriving at any decision. I have myself brought a number of cases before this society upon which I desired an expression of authoritative opinion, opinions that might fix a standard mode of procedure under certain fixed conditions, and I have been disappointed every time. I think that one point in the paper, advising that we might at times raise a question that was unsettled, and discuss and settle it, as far as the opinion of the Odontological Society, or any other society, could settle it, is a good rule of practice to follow, for in that matter we could get a system that would be of great value to ourselves, and to others who do not attend our meetings, but who read the reports of the proceedings in the dental journals. I think it would be a good thing if a series of questions were to be promulgated by the Executive Committee of this society, questions that are to-day unsettled. There are many conditions that present themselves in our practice of which we do not know the causes, and we are uncertain as to the best mode of treatment. I think if such questions were brought before a meeting, time being given for members to prepare themselves for the discussion by careful scientific research, we would advance very materially both as a society and as a profession.

Whether a biennial meeting would be an advantage or not, I do not know. We have now two or three national associations, and I cannot see any advantage that would be gained by multiplying these. I think it advisable to work through the associations we have, or drop these and organize others, rather than form any additional ones. Whether new organizations would be any better than those we have is a question.

The subject of dental colleges is brought up in the paper. This society has a great interest in that question, because the future members of our profession are to come from the dental colleges. How shall students be admitted? under what conditions? what preparation shall we demand from those who seek to enter? and what acquirements shall we demand before the students shall receive their diplomas from these schools? All these are pertinent and important questions. The best dentists in the world are in America, and as poor dentists as are to be found in the world are in this same country. I am firmly of the opinion that a preliminary examination as to education ought to be demanded. It is required nominally now by the dental colleges, but how much it is worth as the requirement exists to-day you can judge as well as I. It ought to be a positive requirement before any student is admitted in a dental college; and the three years' course should be a progressive course. It seems to me ridiculous that a man should be asked to listen to the same lectures for three sessions. A man of good intelligence and application is handicapped from the beginning under the conditions that exist in the colleges to-day. He must attend three years, and the sluggard and the dullard are not required to attend any longer. I think acquirements, and not length of time, should be the standard. It is ridiculous to say that a bright, intelligent, and industrious fellow shall stay in college three years because some dull man requires that term of study. If the one can acquire in twelve months what the other man requires three years to accomplish, he should receive his diploma in twelve months, and not be compelled to wait for the duller or less industrious student.

Now, about this dental club. I am fully in favor of a dental club in New York City, or an organization of dentists in this city that shall own a building, having a room in it, say, of this capacity, and another room for larger meetings, also a reading-room and a good dental library. We have no complete or even good dental library in the United States that is open to the dental public. I suppose the one owned by Dr. A. L. Northrop is as fine a dental library as any in the country, but it is a private library, and although Dr. Northrop is very liberal in allowing access to it, we should have something more public. We ought to have a good museum and one of the most complete laboratories in the country, where original scientific research could be carried on by members. All this would not be difficult to attain. It would cost a good sum of money, but if the spirit that Dr. Bödecker has manifested in his communication, and that shown by Dr. Flower, of Pittsburg, Pa., who also spoke upon the subject, should be followed by a few others, it would not take long to raise the necessary funds. But an enterprise of this kind could be started with very little money under a scheme like this: Suppose such a property as would be required would cost $80,000; the probability is that one-half of that amount could be raised on bond and mortgage at four or four and a half per cent., and the other half, in the shape of a second mortgage bond, should be taken up by members of the club at the same rate of interest, issuing $100 bonds up to the necessary amount. The meeting-room could be rented from time to time, and possibly all the time, which would bring in an income to the club; the dues of members, with the income from rents, should be sufficient to pay the running expenses, and the initiation fees be allowed to accumulate as a sinking fund to pay off the indebtedness. I know of social clubs, a number of them, that have been organized on that basis, within the last few years, and that have not been financial failures. I have no doubt but that the bonds would be very readily taken. I think this club question is well worthy of earnest and immediate consideration.

*Dr. Dwinelle.*—I suppose, Mr. President, that I echo the sentiments of the gentlemen present when I say that the paper itself seems to indicate its own approval; that is to say, the propositions are made in such a way that we necessarily fall in with and approve its suggestions and endorse the whole plan. We have hardly time to discuss the matter at length and in detail, but so far as I am concerned, I approve of what is suggested by the paper at large. I think the time has come when we should make an advance and take a higher ground, a more exalted stand, and look to a broader and more extended future for our profession. I feel, for one, very much obliged to Dr. Atkinson for bringing this matter before us in the lucid and satisfactory way in which he has done it. It seems to have been an open question with us for a long time, only waiting the opportunity, seemingly, for some one to take the final and advanced position that he has taken. Without going into it in detail, I accept and approve generally the plan and system suggested. It goes without argument.

I cannot refrain from alluding to at least one of the propositions in the paper, notwithstanding the lateness of the hour. It seems to me that the great advance which has been made in our profession has created a necessity which is foreshadowed in the series of interrogations in proposition No. 21. We do indeed need "a dental club, organized, equipped with efficient rooms and apparatus and library for clinical, experimental, and literary work, social intercourse, and the entertainment of guests." Such a club would be to us a *home*, where we might meet on common ground, where we could not only investigate and discuss the scientific topics of the day, but where, with a laboratory, embracing all facilities pertaining to art, chemistry, or mechanics, we might in mental effort make new combinations and new discoveries which would never have been attained alone. I believe in this union of purpose whether of the moral, social, intellectual, artistic, or the mechanical departments.

A club of the character referred to would facilitate the "creative good" within us, which ever underlies the spirit of progress; where, under the social range, human nature is at its best, and where, by the magnetism of contact of kindred minds, a thousand things might be discovered for the benefit and amelioration of mankind. A club whose deliberations would become authoritative. I believe in such a club.

*Dr. Meriam.*—Mr. President, I think this paper to-night shows a tendency of the time. There never was in the history of the world, I suppose, a period of greater material advance than the present, and at the same time more "divine dissatisfaction." It is interesting to note that there has been a great awakening of desire among all leaders of thought, presidents of universities, leading preachers in the pulpit, masters in science, and all students of ethics,—that is, of "motives and tendencies" to learn and teach the right way.

"Robert Elsmere," "Looking Backward," "John Ward, Preacher," have all outsold books telling how to make money. It is not strange that this spirit should enter our ranks. We may not be in danger, but the remarks to-night are evidence of the presence of this spirit, and we are asking, "What shall we do to be saved?" or, "How shall we best secure, maintain, and transmit our profession or specialty?"

One of the principles of Professor Agassiz was that the scientist has nothing to do with defending theories; that the duty of the scientist was to discover and place on record, and when he undertook to defend theories he was defending himself. The danger which Professor Agassiz looked forward to in the future of scientists was that they would spend their time in defending doctrines rather than in scientific research. We need societies and journals that give an authorative record of the history of our profession. Much of our trouble has been that we have no such record. This seems to me one of the things that the proposed club could do. It would afford a centre to which students would come not only to conduct experiments and to learn the latest results of the same, but also to learn dental history. Reports would come to an association of that kind, and as the government asks the National Academy of Science to determine questions of a scientific character, so the records of such an institution would furnish positive evidence for the profession.

There is a point in regard to the schools; it seems to me that students should be required to write papers to be read before some member of the faculty conducting a clinical or other conference; questions to be asked by other students, and finally the whole summed up by the member of the faculty having charge. This would be of great value to students. I know how easy it is for young men to drift away after leaving school, simply because they are not kept in hand; and those men would be held, and benefited by the practice of writing during their term of service in school. All society organizations are virtually an acknowledgment of the necessity for consultation; and in consulting over certain difficulties we prepare the material to solve other difficulties. A society is virtually an enlarged consultation. All scientific and professional organizations should be democratic; there should be no special privileges for any one member over another, and there should be no divided interests. "No man can serve two masters," and no man can be loyal to his profession or specialty and be an auxiliary to a dental depot at the same time.

Another thing in connection with this club should be a museum, where we can gather not only pathological specimens, but where also the history of the appliances of our profession may be represented. There are old practitioners passing from the scene who, if such a place were provided, would donate their libraries, instruments, and specimens, and the student could there learn the whole of the past of his profession.

I think also that a journal would properly find its home in such a club. We have certainly had nothing in the way of news of our profession. Visitors from abroad come and go, and very few in the profession know anything about it. There is no organized plan, and very little is possible under our present arrangement. There should also be a directory in connection with such an institution. It seems to me that in a great city like New York it should be no more impossible for a man to have made a new dental instrument or dental engine than it is for a physician to have made a new surgical instrument. I do not know of any profession that has been so liberal or that has recompensed its workmen as our specialty has. The list should also include engravers, draughtsmen, etc.

The syringe which Dr. Kingsley has presented to-night is as well worthy of illustration as any study in microscopy, yet we have omitted such things in our journals in the past. I believe the thing is coming, and I hope we will live to see it.

*Dr. S. E. Davenport.*—I think a few words should be said in commendation of Dr. Atkinson's treatment of the subject of the publication of the transactions of societies. It has seemed to me that some of the most valuable hints are lost because the instruments or methods presented or described are not illustrated, and therefore the few only who are present at a stated meeting and have the opportunity to handle the instruments or appliances can get the idea and receive the benefit. I believe this society is greatly in favor of independent journalism, for it has so placed itself on record unanimously within the past six months, and after publishing its proceedings for many years in the *Dental Cosmos* it has now passed to the INTERNATIONAL DENTAL JOURNAL, the supposed leading independent journal. There will be improvements made in independent journals in time, for they are yet very young. Among those improvements I might mention the necessity for a prompt and business-like attention to copy received from the editors of dental societies, so that the transactions of any particular society may be presented to the dental world as rapidly as they can be prepared. It may be that some journals try to publish the transactions of too many societies, and therefore have not room for all to be promptly served. If that is the case then the journals must be enlarged, or multiplied in number, and the societies apportioned so that, as Dr. Atkinson has said, the matter shall not lose its force by being delayed. Of course, any single society reaches but few dentists if it does not publish its proceedings; its influence depends almost entirely upon what the readers of its transactions find.

*Dr. Meriam.*—Mr. President, one more remark about this proposed dental club, for I think it will come one of these days. I hope that it will be thoroughly guarded so that it cannot be made a hot-bed for tooth crown companies, or anything of that sort. If they are to have introduction there as, in the kindness of our hearts, we have given them introduction heretofore, I cannot imagine anything worse for our specialty.

*Dr. William H. Atkinson.*—I think we should not take counsel of our fears, but of our hopes and aspirations for better things. I think if we are always conjuring up bugaboos, we will always have plenty of them to deal with. We may sometimes nominate the brightest child a bugaboo and divert interest from it, when otherwise it would help on the very purpose of our lives.

What is science? Science means the food for the scientist to feed upon; and it has its analogue in the food of the body,—that is, the various materials that are not exactly in the condition in which the stored energy in them can be appropriated by the body, but need some sort of digestory process. Dentistry has proved to the world that it cannot comfortably subsist upon the *effete* pabulum presented by extant medical and physiological investigations, and in pathological manifestations and therapeutic management. We have been able to reduce polypharmacy pretty nearly to unipharmacy. The time was when physicians' prescriptions contained from five to fifteen elements, with the assumption, on the part of the writers of the prescriptions and the compounders of them, that they comprehended the digestory processes through which these combinations went, and the potency of the remedy as a whole.

What do we need most? Just what will help medicine most, and what would help commerce most. Let each and every department of legitimate human societary arrangement take care of itself; let the dental depots do their best; and if we are in earnest to do the work for each other that we feel the need of being done, and will accept the inspiration of the moment when the responsibility is sprung upon us, inquiring with that degree of earnestness that will not take no for an answer, but seeks a demonstration, then we will rise above all this low ground of fighting figments that we have conjured up, and that are nothing but bugaboos. The difference between our time and that of two hundred years ago is due to concentrated effort. Then men sought to supply their means of cure from individual effort, but it has at last dawned upon us that it is better we should have combination of labor, and that we should have it in the line of clinical teaching, and rid ourselves of the old creed that set limitations to our investigations, and prevent our gaining the real advantage there is in society in any of the departments of life.

The thing we most need to know is how the tissues are nourished. What is nourishment, what is poison, and what is remedy? We want to go behind nearly all that has been recorded on this subject, and secure a syllabus that shall stand to us as the multiplicationtable stands to arithmetic, as a measure by which we can at once pronounce a very fair supposition or hope that such and such will be the result of the administration of such and such remedies or management. There is no place where molecular change is taught, so far as I know, so as to reduce it to a practical presentment that will enable the individuals who deal with remedies to have even confidence in themselves that what they prescribe is legitimately prescribed, and that the result may be foretold. This idea has already found lodgement in the minds of a few noble men, such as Dr. Bödecker and Dr. Flower. You see how active it is when in a nascent state; it is not the *effete* trash that is postulated and recorded as the final truth for all time, but it is the truth of this present time and its necessities. Our ideas of diseased activity have entirely taken a new basis; the old is no longer useful, and we must take that which is fresh and new and obey it and succeed, or follow the old and fail.

The subject is too great and voluminous and profound, and too important for us to ignore any longer. Now the great thing is to lay out a plan. Do it modestly, but do it. Go to work if you know what to do; or, as I say to my pupils, if you do not know what to do, do nothing; wait, or ask counsel from others. Let us pool our knowledges and ignore our fears and our dread of the domination of his satanic Majesty that has ruled us through the old fogy notions that are dead, and ought to have been buried long since, and carrying this idea of having different schools of medicine and different specialties. Medicine should be regarded as a healing art, and anything that comes within that healing art and ministers to those who are suffering is what we want; and we do not care whether we are called medical men or not.

*Dr. Kingsley.*—I was especially requested to make some remarks upon this paper, and the synopsis was sent to me as well as to others. It would take too long for me to attempt to answer each one of those specific points, and besides that, I have not the ability. I have not sufficient interest in some of them to attempt to form an opinion. But there are a few things that I will notice.

"Does private pupilage tend to develop better and more capable operators?" I suppose that every old New England farmer, who has been on a farm from childhood, and lived to seventy or eighty years of age, would answer that there was but one way to learn farming, and that was the way he learned it, and that those fellows from the agricultural colleges, with their new-fangled ideas about chemistry, did not know much about farming; he would say, they ought to go back on a farm and know how to farm first, and then they might go to an agricultural college; but to take an agricultural course before knowing anything about a farm is folly. As I am one of the old school who began in dentistry more than forty years ago, I am something like that old farmer. I feel that private pupilage is one of the best ways of learning dentistry. If you add to that a college course, that is so much clear gain; but no amount of college education in the world can take the place of the practical training of the pupil in a dental office, if thorough and under a competent instructor.

"Private pupilage permits the selection of material, which indicates that certain qualities must be inborn to make a truly professional man possible."

That is simply summing up what I have had to say.

"Charges for infirmary operations have a pernicious influence on the mind of the student, if not responsible for any other evil. It seems proper that every effort should be made to elevate the moral status of the student."

I do not know anything about whether charges for infirmary operations have a pernicious influence or not. It would take me a good while to find out. I would have to make a pretty thorough study of the matter before I could determine conscientiously, from a judicial stand-point, whether it has any such influence. There is a feeling lying back of the whole thing, and it is this: that an infirmary for dental or surgical patients should not impose a tax upon those poor patients who are invited to come there; and I do not think it is just to impose upon them a tax which is equal to the fee which some poor young dentist would charge who is anxious to get a clientele, and is willing to practise very cheap for the sake of making friends and showing what he can do. He argues, "If I do this thing well for this poor person, who cannot pay me anything but the cost of the materials used, he will be likely to recommend me to friends who are able to pay more." I do not think it is quite fair for a dental infirmary to charge as much or more than the young dentist is willing to do the work for. I do not know that it affects the moral status of students.

"Will not a biennial congress, not necessarily international, conducted on an organized, comprehensive plan, work our advancement?"

I do not know. I am inclined to think, as Dr. Jarvie does, that we have societies enough, particularly societies that meet annually. Think of what our First District Society has been doing in the last two or three years! It has held great annual meetings which were attended by representative dentists from other cities and States; in fact, a whole congress; and in addition to that we have the various State societies, with their annual meetings, and the national associations. I think we have entirely too many. It has come to be a decided bore to attend them, and if there were fewer of them I think we would get better results.

I have one criticism to make upon the American Dental Association. I do not like it. I do not go there. I never received any special benefit from it. Dentists go there and divide themselves up into sections, and they spend most of the time in political schemes and smoking. I belonged to a section once. I was the secretary. It had two other officers. That section held big meetings; there was a large attendance. The chairman of the section was there, and the secretary was there; that was all. The rest of the sections, I understood, were conducted in very much the same way. A year or two later, perhaps, when one had time to read the transactions, he might discover something that was offered before some section there. My time has been frittered away in attending meetings of the American Dental Association because I had no axe to grind. I did not want office. If I had wanted office it might have paid me to go. I think it more honorable to be a private, and so I do not go any more. The whole trouble with the American Dental Association is that the originator had a hobby, which was to model it after the American Medical Association, as though we had not independence enough to make our own plans and our own system!

"Cannot a dental club be organized, equipped with sufficient rooms and apparatus, and a library, for clinical, experimental, and literary work, social intercourse, and the entertainment of guests?"

The way it has started this evening it can be. But how many times among dentists have we seen a thing started, not like a rocket, but started from the top and come down; started with a splurge, and that was all there was of it. I would like to see a dental club in this city. I belong to several clubs now. There are certain associations in them, certain gentlemen whom I like to meet, and that is why I belong to those clubs. I affect once in a while the artistic; and I like to belong to a club where there are artists, and where I can make myself think that I am one of them. I would like to belong to an authors' club. There is one, but they won't let me in because I am not an author by vocation. I am an author, but not an author by vocation. I am a member of several clubs, but in none of them do I have the blessed opportunity of enjoying the society of my *confrères* in dentistry. Therefore, on some accounts, I would like to see a dental club established where I could drop in and chat without being obliged to talk to the universe. It is one of the most embarrassing things for me to stand up and talk to the universe. When I get the report of my remarks I put a pen through a good part of them, and they print about one-fourth of what I say. In a club where one's remarks are not taken down, if I feel like speaking out very strong I can speak; but one cannot do that when it is going into print.

It is quite possible that a dental club can be established and made a success, provided the attempt is not also made to run a bar and restaurant. The bar and restaurant have sunk more clubs in this city than anything else. Perhaps some of us would like a bar and restaurant, but if the club is to succeed the bar and restaurant should be on the outside. So much for the synopsis.

Now, there have been some remarks made this evening that galled me. I am speaking plain, old-fashioned English, Anglo-Saxon. Hardly a speaker has been on his feet that has not talked about "our specialty," and in the next breath about "our profession." One gentleman, in his remarks about independent dental journalism, said, what I did not know until this evening, that this society has committed itself unanimously to independent dental journalism. Now, as near as I can find out, the independent dental journalism which this society has committed itself to, if it has at all, is made up of the veriest twaddle. That is plain English. It is nothing but twaddle. Independent of what? Independent of a company of publishers that are able to publish a thoroughly creditable journal? Is that independent dental journalism? Not according to my idea at all. Independent dental journalism should represent dentistry in its independence. I tell you, gentlemen, that what is now doing more to menace the integrity of the dental profession—I use the word profession—than all else is not the men who are preparing materials and furnishing goods for dentists' use, but it is the class of men who are trying to attach dentistry to another profession. I got on board the "Augusta Victoria" at Hamburg, last October, and I was surprised as well as gratified to see one of my colleagues coming on board at Southampton. The gentleman, I found, was one of the directors of one of the journals we have heard about. I asked him who controlled the journal, and he made me an answer. As we dropped into further conversation upon the subject, I made some remarks, referring to the title of their journal being a misnomer; that it was not independent at all; that it did not stand up for dentistry as dentistry, but stood for dentistry as something else; and I asked him why not start out plainly and boldly with real independent journalism, and stand up for dentistry as an independent profession. He could give no reason.

Now, what does all this talk about a club point to? The desire is to have a dental club. Can it not be seen that dentists are all the while making themselves more and more pronounced as a separate profession, and yet for fear they won't be recognized as a specialty they do not dare to stand up for themselves and say, "We are dentists and we are proud of being dentists." Our main object should be to make dentistry the best dentistry that can be made, without being anxious to have it tacked on to the end of something else. We *are* doing that now; and this movement for the formation of a dental club only shows that the tendency is in that direction, in spite of a class of men who would drag it down.

*Dr. Jarvie.*—Mr. President, I want to see some recognition taken of this letter of Dr. Bödecker; and I move you that the secretary be directed to acknowledge the receipt of the letter, and that the communication itself be referred to the Executive Committee, together with the proposition of Dr. Flower.

Motion carried.

On motion of Dr. Dwinelle, a vote of thanks was tendered to the essayist of the evening, Dr. Charles B. Atkinson, for his very able paper.

Adjourned.

S. E. Davenport, D.D.S., M.D.S.,
*Editor New York Odontological Society.*

## Figures and Tables

**Figure f1:**